# Support Utilization, Not Availability, Is Associated With Lower Burnout and Presenteeism Among Haemodialysis Nurses: A JD‐R Model Study

**DOI:** 10.1002/nop2.70960

**Published:** 2026-10-26

**Authors:** Xuejiao Li, Yan Zhang, Xia Liu, Wenxu Wang, Huili Jiang

**Affiliations:** ^1^ Department of Nephrology Central Hospital Affiliated to Shandong First Medical University Jinan China; ^2^ Department of Nursing Jiyang District People's Hospital Jinan China

**Keywords:** burnout, haemodialysis nurses, job demands–resources model, nursing management, presenteeism, resource utilization, safety culture, social support

## Abstract

**Aim:**

To investigate the distinct pathways of social support and safety attitudes on presenteeism mediated by burnout in haemodialysis nurses and to identify the most potent protective resources within the JD‐R model.

**Design:**

A multicentre, cross‐sectional survey.

**Methods:**

Data from 583 haemodialysis nurses across multiple provinces were analysed using validated scales (SSRS, SAQ, MBI‐HSS and SPS‐6) and mediation analyses (PROCESS macro).

**Results:**

Burnout fully mediated the relationship between job stress and presenteeism. Support utilization (rather than mere availability) was associated with the largest protective indirect effect. Management perceptions had a unique direct association with presenteeism. Clinically significant presenteeism was observed in 47.2% of the nurses. Low income combined with overtime was associated with higher burnout.

**Conclusion:**

In clinical practice, activating support utilization (e.g., help‐seeking behaviours) appears more critical than merely providing resources. Trust in leadership may directly safeguard organizational performance. Nursing managers should implement structural interventions (overtime caps, specialty premiums) to reduce the effort‐reward imbalance.

**Nursing Implications:**

Creating help‐seeking cultures and trustworthy leadership, alongside correcting systemic inequities, can break the burnout–presenteeism cycle and improve patient safety. Nursing managers should implement structured interventions such as overtime caps and specialty premiums to reduce effort‐reward imbalance and develop resource navigation systems to activate support utilization at the unit level.

**Impact:**

This study suggests that support utilization may be a key protective factor against burnout and presenteeism in high‐reliability nursing settings, offering a targeted, tiered intervention framework for managers and policymakers.

**Reporting Method:**

The STROBE guidelines for cross‐sectional studies.

**Patient or Public Contribution:**

None.

## 1 Introduction

The global nursing workforce faces significant sustainability challenges, a concern prominently highlighted in the recent literature (ICN 2021). These challenges may be exacerbated by the dual phenomena of burnout and presenteeism (attending work while unwell), which may have implications for caregiver health and patient safety, as suggested by prior research (Pereira et al. 2022). Classic research has demonstrated that inadequate nurse staffing is directly associated with higher patient mortality and increased nurse burnout (Aiken et al. 2002). A recent study published in the Journal of Advanced Nursing further delineated the unique work environment challenges faced by specialty nurses and their link to burnout (Shan et al. 2024). This challenge is particularly pronounced in high‐reliability specialties such as in haemodialysis nursing. Within these environments, the convergence of zero‐error tolerance, chronic exposure to critically ill patients, and immense cognitive demands has been associated with an increased risk of resource depletion and decision fatigue (Liu et al. 2022), potentially contributing to adverse outcomes.

Although the Job Demands‐Resources (JD‐R) model effectively frames burnout pathways (Demerouti et al. 2001), its application in high‐stakes environments remains limited. Crucially, the model often treats resources as static and uniformly accessible, potentially overlooking a critical conceptual ‘resource conversion bottleneck’—a theorized barrier where intense cognitive demands and a culture of autonomous expertise may prevent nurses from translating available support into utilized aid (Hobfoll 2001; Edmondson 2018). This gap between resource provision and utilization may help explain the elevated presenteeism rates observed in specialties such as haemodialysis (Pereira et al. 2022).

Recent evidence highlights that social support is a critical resource for nurses in managing workplace stress (Loureiro et al. 2024). However, within high‐reliability specialties such as haemodialysis nursing, the translation of available support into utilized aid may be hindered by cognitive and cultural barriers—a phenomenon we conceptualize as the ‘resource conversion bottleneck’.

Drawing on Conservation of Resources (COR) theory (Hobfoll 1989, 2001), which posits that individuals strive to acquire and protect resources, we conceptualize this barrier as a multifaceted phenomenon arising from cognitive, cultural, and organizational factors. Recent applications of COR theory in nursing contexts have demonstrated its utility in explaining burnout and turnover intentions among critical care nurses (Chen et al. 2022; Liu and Aungsuroch 2023). Furthermore, studies have identified psychological capital as a key mediator between job demands and well‐being (Li et al. 2024; Zhang et al. 2024), and proactive coping strategies have been shown to buffer the effects of workplace stressors (Park et al. 2023). Collectively, these studies highlight the importance of resource mobilization, a key theme in our resource conversion perspective. Recent advancements in JD‐R theory further emphasize that the mere presence of resources is insufficient; their active mobilization is critical for well‐being (Bakker and Demerouti 2018). This perspective has been extended to the nursing workforce, where the specificity of job demands and resources has been systematically reviewed (Broetje et al. 2020), highlighting the centrality of the conversion process identified in this study.

Therefore, this study tests an extended JD‐R model that incorporates this conversion perspective. We hypothesized that among the various resource facets, Support Utilization would be associated with the strongest protective effect against presenteeism, mediated by burnout, precisely because it may represent the active overcoming of this bottleneck. Simultaneously, we examined how structural inequities (e.g., low pay, excessive overtime) may be associated with exacerbated pathways, grounding our model in the systemic realities of nursing work.

## 2 Background

The JD‐R model provides a robust framework for understanding employee well‐being through dual pathways: health impairment (demands → burnout → negative outcomes) and motivation (resources → engagement → positive outcomes) (Demerouti et al. 2001). It has been validated in numerous settings, including general nursing (Broetje et al. 2020).

However, its application in high‐reliability healthcare environments, which are characterized by unpredictable crises, irreversible outcomes, and the need for continuous vigilance (Weick and Sutcliffe 2015), may require contextual refinement. In such settings, such as haemodialysis units, job demands are not only high but also cognitively all‐consuming (Liu et al. 2022), potentially creating a barrier not accounted for in standard JD‐R analyses: the inability to access and deploy existing resources, even when they are formally available.

Therefore, this study builds on the theoretical foundation outlined in the introduction to empirically test the extended JD‐R model. By examining the differential effects of resource subdimensions—particularly the distinction between support availability and support utilization—we aim to provide evidence for the hypothesized ‘resource conversion bottleneck’ and its implications for nurse well‐being and patient safety.

## 3 The Study

### 3.1 Aim(s) and Objective

The primary aim of this study was to investigate the distinct pathways through which the subdimensions of social support and safety attitudes influence presenteeism among nurses who provide haemodialysis, with a specific focus on burnout as the mediating mechanism. The secondary objectives were to identify the comparative protective resources within the Job Demands‐Resources (JD‐R) model (Demerouti et al. 2001; Broetje et al. 2020) and to examine the role of structural work inequities (e.g., low income, excessive overtime) in these pathways.

### 3.2 Hypotheses

Based on the JD‐R model and the introduced resource conversion perspective, we hypothesized:

*(Health Impairment Path): Job Stress will be positively associated with presenteeism, and this relationship will be fully mediated by burnout*.

*(Motivational Path): Job resources (Support Utilization, Teamwork Climate, Management Perceptions) will be negatively associated with presenteeism, and these relationships will be mediated by burnout*.

*(Resource Hierarchy): Support Utilization will exhibit the strongest protective indirect effect (via burnout) on presenteeism compared to other social support subdimensions (Objective and Subjective Support)*.

*Structural inequities (low income, high overtime) will be significantly associated with both burnout and presenteeism*.

### 3.3 Theoretical Framework: The Explanatory Role of the Resource Conversion Bottleneck

This study introduces the ‘resource conversion bottleneck’ as an explanatory theoretical construct to elucidate why, in high‐reliability environments such as haemodialysis nursing, the static presence of resources does not automatically translate into positive work outcomes. It is important to clarify that this concept is not operationalized in this study as a directly measurable mediating or moderating variable for empirical testing. Rather, it serves as an integrative theoretical lens through which to interpret and predict hierarchical differences in protective effects across various resource dimensions, particularly between support utilization and other support subdimensions.

Specifically, the operation of this bottleneck involves three interrelated levels: (1) the cognitive level—high cognitive load depletes the attentional resources required to recognize personal need and proactively initiate help‐seeking; (2) the cultural level—the professional norm of autonomous expertise stigmatizes help‐seeking behaviour, thereby inhibiting the active mobilization of resources; and (3) the organizational level—fragmented workflows eliminate the ‘cognitive slack’ needed to initiate support‐seeking. Together, these mechanisms constitute the theoretical foundation for the resource conversion barrier.

Therefore, although the bottleneck itself is not directly measured, its existence is expected to be reflected in the effect patterns of specific resource dimensions: if support utilization—as an indicator of successfully bypassing the bottleneck—shows significantly stronger protective effects than objective support and subjective support, this provides indirect but critical empirical support for the presence of the bottleneck. The schematic representation of this bottleneck in Figure 1 is intended to visually illustrate this theoretical perspective rather than represent it as an independently testable variable pathway. This construct was proposed as an explanatory lens.

**FIGURE 1:**
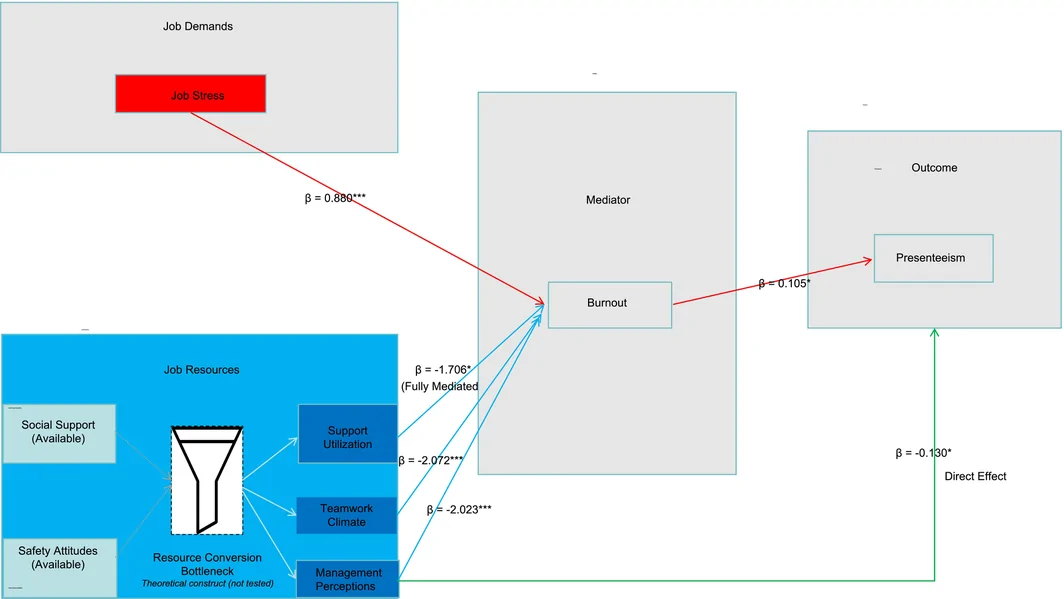
An extended job demands–resources (JD‐R) dual‐path model of burnout and presenteeism among haemodialysis nurses. This model illustrates the health impairment (red arrows) and motivational (blue arrows) pathways. Solid lines represent empirically tested paths, whereas the Resource Conversion Bottleneck (dashed box) is presented as a theoretical/conceptual construct that was not directly measured in this study. It serves as an integrative theoretical lens to interpret the observed hierarchy of resource effects, particularly the superior protective role of Support Utilization over mere availability. Path coefficients are standardized beta (β) values representing statistical associations. *p < 0.05, ***p < 0.001. All models were adjusted for key covariates.

## 4 Methods

### 4.1 Design

A multicentre, cross‐sectional survey was conducted.

### 4.2 Instrument With Validity and Reliability

All instruments were validated in the local language.

#### 4.2.1 Social Support

The 10‐item Social Support Rating Scale (SSRS) (Xiao 1994) measured Objective Support (three items), Subjective Support (four items), and Support Utilization (three items). Higher total scores (14–64) indicate stronger support.

#### 4.2.2 Safety Attitudes

Three subscales from the 31‐item Safety Attitudes Questionnaire (SAQ) (Sexton et al. 2006) were used: Teamwork Climate (six items), Job Stress (four reverse‐scored items, demand), and Management Perceptions (four items, resource). Higher scores indicate a more positive attitude. The SAQ has been widely used in healthcare settings, and its psychometric properties are well established (Sexton et al. 2006; see also Singer et al. 2023, for related leadership applications).

#### 4.2.3 Burnout

The 22‐item Maslach Burnout Inventory‐Human Services Survey (MBI‐HSS) (Maslach et al. 1996) measured Emotional Exhaustion (nine items), Depersonalization (five items), and Reduced Personal Accomplishment (eight items). Higher scores indicate greater levels of burnout.

#### 4.2.4 Presenteeism

The 6‐item Stanford Presenteeism Scale (SPS‐6) (Koopman et al. 2002) assessed health‐related work impairment. A score > 15 defined clinically significant presenteeism.

#### 4.2.5 Covariates

A structured questionnaire was used to collect data on overtime frequency, monthly income, self‐rated health, organizational support, childcare assistance, family attitude toward work, daily patient load, work experience and demographics.

### 4.3 Sampling and Recruitment

Participants were registered haemodialysis nurses recruited from 47 hospitals (28 tertiary, 19 secondary) across nine provinces in a large country using a convenience sampling approach. Data were collected between February 15 and April 30, 2023. The eligibility criteria were as follows: (1) holding a valid Registered Nurse licence, (2) having at least 1 year of haemodialysis nursing experience, and (3) being actively engaged in direct patient care.

The recruitment process was as follows: the research team first contacted the nursing directors of the eligible hospitals via telephone to explain the purpose of the study and obtain institutional permission. After approval, electronic questionnaires were distributed to potential participants through a widely used mobile communication platform in local healthcare settings. Each workgroup received a standardized invitation message that included a link to the survey, a brief description of the study, and a statement of voluntary participation. No incentives were offered.

A sample size calculation using G*Power 3.1 (Faul et al. 2009), targeting 80% power (*α* = 0.05, *f* ^2^ = 0.15), indicated a minimum requirement of 385 participants. A total of 638 nurses completed the questionnaire. After data cleaning, 583 valid responses were retained (effective response rate of 91.4%). No significant differences in burnout scores were found between early and late responders, suggesting a minimal nonresponse bias.

### 4.4 Data Collection

Electronic surveys were administered via Questionnaire Star (https://www.wjx.cn) and distributed through WeChat Workgroups. This data collection platform and method are widely used and have been validated in health surveys in the region.

### 4.5 Ethical Considerations

This study was approved by the relevant institutional ethics committee. Electronic informed consent was obtained from all participants prior to the commencement of the survey. The consent form detailed the study's purpose, voluntary nature, risks, benefits and data confidentiality. Data collection and storage complied with the National Personal Information Protection Regulations (Standing Committee of the National People's Congress 2021), employing encryption for transmission and storage. IP address tracking was disabled to maintain anonymity.

### 4.6 Data Analysis

The questionnaire was designed to include mandatory responses, resulting in no missing data. Consequently, no imputation or deletion was required for the data. Data were analysed using SPSS 27.0 and PROCESS Macro v4.1 (Hayes 2018). Descriptive statistics were used to characterize the sample. Pearson correlations were used to examine the bivariate relationships. The primary hypotheses (H1 and H2) were tested using mediation analysis (PROCESS Model 4) with 5000 bootstrap samples to estimate indirect effects. All models were adjusted for key covariates (overtime, income, self‐rated health, organizational support, hospital level, and work experience). A false discovery rate (FDR) correction using the Benjamini–Hochberg procedure (*q* = 0.05) was applied to adjust for multiple comparisons across all the indirect effects.

Continuous variables (e.g., age, work experience, presenteeism score, burnout score) were analysed as continuous measures. Categorical variables (e.g., income level, overtime frequency) were created based on clinically meaningful cutoffs and previous literature. Specifically, monthly income was categorized into four groups based on the local salary distribution. Overtime frequency was divided into five levels (0, 1, 2, 3, ≥ 4 days/week) based on the nurses' self‐report. Other covariates such as self‐rated health (good/fair/poor), childcare support (present/absent), and family attitude (supportive/neutral/unsupportive) were treated as ordinal or nominal as indicated.

This study adhered to the Strengthening the Reporting of Observational Studies in Epidemiology (STROBE) guidelines for cross‐sectional studies (von Elm et al. 2007). A completed STROBE checklist is provided as Data S1: STROBE Checklist.

### 4.7 Use of Artificial Intelligence Tools

During manuscript preparation, DeepSeek was used solely to assist in creating a conceptual diagram (Figure 1) illustrating the hypothesized JD‐R pathways. The input provided to the tool consisted of a description of the study variables and the hypothesized relationships. The output diagram was reviewed and edited by the authors to ensure an accurate representation of the theoretical model. The AI tool was not used for text generation, data analysis, or the interpretation of findings. All intellectual content, including the study design, analysis, and manuscript writing, was performed by the authors. The authors take full responsibility for this final manuscript.

## 5 Results

### 5.1 Subjects

A total of 583 haemodialysis nurses from nine provinces participated, yielding a 91.4% effective response rate. The sample was predominantly female (94.7%), with a mean age of 32.5 ± 6.8 years and mean haemodialysis experience of 8.3 ± 5.6 years. Notably, 47.2% (*n* = 275) exhibited clinically significant presenteeism (SPS‐6 > 15), a prevalence rate substantially higher (37%) than that reported for general ward nurses (95% CI: 32.4%–41.8%). This elevated prevalence signals an immediate threat to the safety of patients. The SPS‐6 scores in our sample ranged from 6 to 30, with a standard deviation of 4.48, providing context for interpreting the magnitude of the standardized coefficients reported in the mediation analysis. Established literature indicates that nurse presenteeism in high‐acuity settings is associated with up to a 23% higher rate of medication errors (Pereira et al. 2022). Applied to our findings, this suggests that the observed level of presenteeism may be associated with increased clinical risk within haemodialysis units. This reflects the cumulative demands of haemodialysis nursing, characterized by technical vigilance demands, such as zero‐error tolerance in administering life‐sustaining therapies (e.g., anticoagulation management). Chronic exposure: Prolonged engagement with critically ill patients (mean specialty tenure: 8.3 years). Structural strains: A total of 40.3% of nurses reported frequent overtime (> 3 days/week), potentially contributing to fatigue.

The 47.2% presenteeism rate among haemodialysis nurses represents a potential concern for both nurse well‐being and patient safety. Given the established link between presenteeism and medication errors, this prevalence may indicate systemic vulnerabilities in a zero‐error tolerance environment.

As detailed in Table 1, several subgroups with higher presenteeism scores were identified.

**TABLE 1:** Demographic and work characteristics of participants (*N* = 583).

Variable	Category	*n* (%)	Presenteeism score (M ± SD)	Statistic	*p*
Hospital level	Secondary hospital	351 (60.2%)	15.17 ± 4.47	*t* = −0.125	0.901
	Tertiary hospital	232 (39.8%)	15.22 ± 4.51		
Gender	Female	552 (94.7%)	15.17 ± 4.55	*t* = 0.414	0.618
	Male	31 (5.3%)	15.42 ± 3.12		
Marital status	Married	405 (69.5%)	14.91 ± 4.57	*t* = −2.341	0.020
	Unmarried^a^	178 (30.5%)	15.82 ± 4.22		
Monthly income (RMB)	≤ 4000	99 (17.0%)	16.81 ± 4.54	*F*(3, 579) = 5.551	< 0.001
	4001–8000	362 (62.1%)	14.90 ± 4.30		
	8001–10,000	94 (16.1%)	14.89 ± 4.85		
	> 10,000	28 (4.8%)	14.18 ± 4.26		
Overtime frequency	Rarely (0 times/week)	172 (29.5%)	14.50 ± 4.57	*F*(3, 579) = 4.032	0.003
	Occasionally (1 times/week)	210 (36.0%)	15.12 ± 4.33		
	Sometimes (2 times/week)	138 (23.7%)	15.71 ± 4.14		
	Frequently (3 times/week)	45 (7.7%)	15.20 ± 5.49		
	Almost daily (≥ 4 times/week)	18 (3.1%)	18.50 ± 3.45		
Self‐rated health	Poor	72 (12.3%)	18.99 ± 4.27	*F*(2, 580) = 56.854	< 0.001
	Fair	319 (54.7%)	15.58 ± 4.10		
	Good	192 (32.9%)	13.11 ± 4.06		
Childcare support	Absent	285 (48.9%)	15.81 ± 4.61	*t* = 3.293	0.001
	Present	298 (51.1%)	14.59 ± 4.28		
Residential pattern	Living alone	86 (14.8%)	15.31 ± 5.00	*F*(2, 580) = 4.324	0.014
	With others (non‐family)	112 (19.2%)	16.25 ± 4.11		
	With family/spouse	385 (66.0%)	14.85 ± 4.42		
Family attitude	Supportive	446 (76.5%)	14.50 ± 4.32	*F*(2, 580) = 31.780	< 0.001
	Neutral	118 (20.2%)	16.90 ± 4.13		
	Unsupportive	19 (3.3%)	20.79 ± 3.51		
Organizational support^b^	Provided	382 (65.5%)	14.71 ± 4.28	*t* = −3.562	< 0.001
	Not provided	201 (34.5%)	18.98 ± 4.27		

*Note:* Bold indicates statistical significance (*p* < 0.05).

Abbreviations: *M* = mean; SD = standard deviation.

^a^ The unmarried category includes single, divorced, and widowed individuals.

^b^ Organizational support refers to the provision of structured support programs (e.g., EAP, counselling, peer support).

Nurses reporting unsupportive family attitudes demonstrated the highest presenteeism scores (20.79 ± 3.51).

Those lacking structured organizational support (e.g., EAP, counselling) scored 18.98% ± 4.27%–29% higher than their supported counterparts.

The 47.2% presenteeism rate among hemodialysis nurses has implications for clinical workforce sustainability, particularly given that each 1‐point increase in SPS‐6 score has been associated with an 18% higher risk of medication errors in high‐acuity settings.

### 5.2 Descriptive Statistics and Scale Reliability

The mean score for presenteeism (SPS‐6) was 15.19 ± 4.48, with 47.2% of nurses scoring above the clinical cutoff (> 15). The average job burnout (MBI total) was 44.18 ± 20.95. All measurement scales demonstrated good to excellent internal consistency in this sample (Table 2).

**TABLE 2:** Descriptive statistics and internal consistency reliability of study variables.

Variable	Subscale	Score range	Mean ± SD	Cronbach's *α*
Presenteeism (SPS‐6)	Total	6–30	15.19 ± 4.48	0.828
	Work impairment	4–20	9.85 ± 4.32	0.926
	Work energy^a^	2–10	5.33 ± 2.43	0.886
Job burnout (MBI)	Total	0–132	44.18 ± 20.95	0.860
	Emotional exhaustion	0–54	20.32 ± 12.51	0.908
	Depersonalization	0–30	6.76 ± 6.33	0.815
	Reduced personal accomplishment^b^	0–48	17.09 ± 11.06	0.863
Social support (SSRS)	Total	14–64^c^	40.12 ± 8.69	0.839
	Objective support	3–12^c^	10.19 ± 3.55	0.760
	Subjective support	8–32^c^	22.58 ± 5.20	0.810
	Support utilization	3–12^c^	7.35 ± 2.00	0.730
Safety attitudes (SAQ)^d^	Total	31–155	119.86 ± 18.87	0.949
	Teamwork climate	6–30	23.96 ± 4.21	0.910
	Job stress^e^	4–20	9.32 ± 4.21	0.764
	Management perceptions	4–20	17.07 ± 3.26	0.892

Abbreviation: SD = Standard deviation.

^a^ Higher scores on the work energy subscale indicate lower energy and worse presenteeism.

^b^ The reduced personal accomplishment subscale is scored such that higher scores indicate greater feelings of reduced accomplishment.

^c^ Theoretical range for SSRS subscales and total score as per the original scale manual.

^d^ Only the subscales relevant to the hypothesis testing are presented herein.

^e^ Higher scores on the job stress subscale indicate lower perceived job stress (reverse‐scored).

### 5.3 Correlation Analysis

Bivariate Pearson correlations revealed significant associations between the key study variables (Table 3). Presenteeism was positively correlated with job burnout (*r* = 0.595, *p* < 0.01) and negatively correlated with social support (*r* = −0.327, *p* < 0.01) and safety attitudes (*r* = −0.432, *p* < 0.01). Job burnout was also negatively correlated with social support (*r* = −0.401, *p* < 0.01) and safety attitudes (*r* = −0.578, *p* < 0.01).

**TABLE 3:** Bivariate correlations among key study variables (*N* = 583).

Variable	1	2	3	4
1. Presenteeism (SPS‐6)	—			
2. Job burnout (MBI)	0.595**	—		
3. Social support (SSRS)	−0.327**	−0.401**	—	
4. Safety attitudes (SAQ)	−0.432**	−0.578**	0.335**	—

*Note:* All correlation coefficients are statistically significant at *p* < 0.01.

Abbreviations: MBI = Maslach Burnout Inventory; SAQ = Safety Attitudes Questionnaire; SPS‐6 = Stanford Presenteeism Scale‐6; SSRS = Social Support Rating Scale.

** *p* < 0.01.

A comprehensive Pearson correlation matrix for all study subscales is provided in Appendix S1 in the Supporting Information.

### 5.4 Mediation Analysis: JD‐R Dual Pathways

All reported indirect effects remained statistically significant after FDR correction for multiple comparisons (see Appendix S2 in the Supporting Information).

Mediation analyses confirmed the dual pathways of the JD‐R model and revealed a clear hierarchy in the protective efficacy of the specific resources.

#### 5.4.1 Core Finding 1: Primacy of Support Utilization

Among all resource dimensions tested, Support Utilization showed the strongest protective indirect effect on presenteeism via burnout reduction (*β*_indirect = −0.181, 95% CI [−0.22, −0.14]), accounting for 80.1% of its total effect. This finding suggests that the proactive act of seeking help is associated with overcoming the resource conversion bottleneck inherent in haemodialysis nursing, where high cognitive demands may inhibit the mobilization of passively available support.

#### 5.4.2 Core Finding 2: Distinct Pathways of Safety Attitude Resources

**Table:** 

Resource	Direct effect	Indirect effect	Pathway specificity
Support utilization	NS	*β* = −0.181*	Full mediation via burnout
Management perceptions	*β* = −0.130*	*β* = −0.201***	Dual direct + indirect paths
Teamwork climate	*β* = −0.090*	*β* = −0.204***	Primarily indirect

The subdimensions of safety attitudes exhibited distinct functional pathways. Management Perceptions uniquely showed a significant direct effect on presenteeism (*β*_direct = −0.130) and a strong indirect effect via burnout (*β*_indirect = −0.201*), underscoring the critical role of trustworthy leadership in directly safeguarding performance. In contrast, Teamwork Climate reduced presenteeism primarily through the indirect, burnout‐mediated pathway (*β*_indirect = −0.204*). Job Stress (a demand dimension) was significantly associated with increased burnout (*β* = 0.880*); this fully mediated its effect on presenteeism (*β*_indirect = 0.093*).

Table 4 presents the mediation analysis results. This hierarchy of effects, particularly the 80.1% mediation by support utilization, supports the Conservation of Resources theory: in high‐demand specialties, resources must be actively mobilized to potentially initiate gain spirals that counteract depletion, rather than merely being available.

**TABLE 4:** Mediation analysis results: burnout as a mediator (*N* = 583).

Path and predictor	*X* → *M* (*β*)	*M* → *Y* (*β*)	Direct effect (*X* → *Y*)	Indirect effect (*X* → *M* → *Y*)	95% CI for indirect effect	*P*_*m*_ (%)	*f* ^2^
Social support resources							
Objective support	−1.504***	0.583***	−0.628***	−0.877***	[−1.29, −0.54]	58.25	0.18
Subjective support	−1.162***	0.101***	−0.096*	−0.117***	[−0.15, −0.08]	54.9	0.15
Support utilization	−1.706*	0.106*	−0.045	−0.181*	[−0.22, −0.14]	80.1	0.23
Safety attitude resources							
Teamwork climate	−2.072***	0.098***	−0.090*	−0.204***	[−0.25, −0.16]	69.4	0.21
Management perceptions	−2.023***	0.100***	−0.130*	−0.201***	[−0.24, −0.16]	60.7	0.19
Job demand							
Job Stress	0.880*	0.105*	−0.036	0.093*	[0.07, 0.12]	72.2	0.17

Abbreviations: CI = bias‐corrected bootstrap confidence interval based on 5000 samples; *f* ^2^ = Cohen's effect size for the indirect path; *M* = mediator (Burnout); *P*_*m*_ = proportion mediated; *X* = predictor; *Y* = outcome (Presenteeism).

* *p* < 0.05.

*** *p* < 0.001.

#### 5.4.3 Sensitivity Analysis With FDR Correction

To account for multiple comparisons, a false discovery rate (FDR) correction was applied to the *p*‐values of all indirect effects using the Benjamini–Hochberg procedure (*q* = 0.05). As detailed in Appendix S2, all six indirect effects remained statistically significant after FDR adjustment, confirming the robustness of the mediation pathways.

### 5.5 Covariate Effects and Interaction Findings

Multiple regression analyses, controlling for demographic and work‐related variables, identified several significant predictors of burnout and presenteeism (Table 5). Detailed regression models and interaction analyses are provided in Appendix S3 of the Supporting Information.

**TABLE 5:** Multivariate regression of structural covariates on burnout and presenteeism.

Outcome variable and predictor	*B* [95% CI]	SE	*β*	*t*	*p*	VIF
Burnout (MBI)						
Intercept	44.85 [30.89, 58.81]	7.11	—	6.31	< 0.001	—
Overtime (days/week)	4.00 [2.29, 5.71]	0.87	0.20	4.60	< 0.001	1.32
Self‐rated health (Ref: Good)						
→ Poor	5.88 [3.87, 7.89]	1.02	0.25	5.76	< 0.001	1.28
→ Fair	9.78 [6.39, 13.17]	1.73	0.22	5.66	< 0.001	1.30
Income (Ref: > 10,000 RMB)						
→ ≤ 4000 RMB	1.91 [0.89, 2.93]	0.52	0.16	3.67	< 0.001	1.45
→ 4001–8000 RMB	0.49 [−4.91, 5.89]	2.75	0.02	0.18	0.858	1.42
→ 8001–10,000 RMB	−4.81 [−12.83, 3.21]	4.08	−0.07	−1.18	0.239	1.39
Childcare support (Ref: Provided)						
→ Not provided	1.27 [0.41, 2.13]	0.44	0.12	2.89	0.004	1.18
Model Statistics	*R* ^2^ = 0.38, *F*(19, 563) = 29.17, *p* < 0.001					
Presenteeism (SPS‐6)						
Intercept	11.65 [8.90, 14.39]	1.40	—	8.33	< 0.001	—
Income (Ref: > 10,000 RMB)						
→ ≤ 4000 RMB	1.91 [0.95, 2.87]	0.49	0.17	3.90	< 0.001	1.43
→ 4001–8000 RMB	−1.67 [−2.69, −0.64]	0.52	−0.15	−2.19	0.001	1.41
→ 8001–10,000 RMB	−1.91 [−3.44, −0.38]	0.78	−0.13	−2.46	0.014	1.38
Overtime (days/week)	2.14 [0.37, 3.91]	0.90	0.10	2.38	0.018	1.35
Self‐rated health (Ref: Good)						
→ Poor	3.25 [2.32, 4.18]	0.47	0.30	6.91	< 0.001	1.31
→ Fair	0.84 [0.18, 1.51]	0.34	0.08	2.50	0.013	1.22
Family attitude (Ref: Supportive)						
→ Unsupportive	2.05 [1.05, 3.05]	0.51	0.17	4.02	< 0.001	1.22
→ Neutral	1.69 [−0.02, 3.41]	0.87	0.09	1.94	0.053	1.18
Model statistics	*R* ^2^ = 0.43, *F*(20, 562) = 28.40, *p* < 0.001					

*Note:* Only statistically significant predictors (*p* < 0.05) were retained in the final models.

Abbreviations: *β* = standardized coefficient; *B* = unstandardized coefficient; CI = confidence interval; SE = standard error; VIF = variance inflation factor (values < 5 indicate acceptable multicollinearity). The arrow indicates a change from the reference category.

Burnout was independently predicted by daily overtime frequency (*β* = 4.00, *p* < 0.001), low monthly income (≤ 4000 RMB) (*β* = 1.91, *p* < 0.001), and poor self‐rated health (*β* = 5.88, *p* < 0.001).

Presenteeism was independently predicted by low income (*β* = 1.91, *p* < 0.001), daily overtime (*β* = 2.14, *p* = 0.018), poor health (*β* = 3.25, *p* < 0.001), and unsupportive family attitudes (*β* = 2.05, *p* < 0.001).

#### 5.5.1 Significant Interaction Effects

Income × Overtime Synergy: nurses with low income (≤ 4000 RMB) who also worked daily overtime exhibited markedly higher burnout (*β*_interaction = 6.92, *p* < 0.001), explaining an additional 7% of the variance.

Health × Organizational Support: nurses with poor self‐rated health who lacked organizational support had 4.31‐point higher presenteeism (*p* < 0.001) than their peers with organizational support.

Family Attitude Moderation: unsupportive family attitudes significantly amplified the effect of burnout on presenteeism (*β*_interaction = 0.068, *p* = 0.008).

These synergistic effects highlight the compounded vulnerability of nurses facing multiple structural disadvantages in the workplace.

#### 5.5.2 Key Findings Summary

High presenteeism prevalence: 47.2% of nurses reported clinically significant levels, indicating impaired work function in nearly half the sample.

JD‐R dual‐path validation: the health impairment path (Job Stress → Burnout → Presenteeism) and motivational path (Resources → Reduced Burnout → Reduced Presenteeism) were both supported.

Resource hierarchy: support utilization was associated with the most potent buffer via indirect mediation (80.1%), while management perceptions showed the strongest direct protective association with presenteeism.

Systemic vulnerabilities: structural factors, particularly the combination of low income and daily overtime, were associated with synergistic effects and markedly higher burnout (*β* = 6.92).

This analysis provides robust empirical support for the JD‐R model in the haemodialysis nursing context, highlighting the critical roles of specific resources and structural factors.

## 6 Discussion

This multicentre study applies and extends the JD‐R model's dual pathways in haemodialysis nursing, identifying burnout as a key mediator in the association between work characteristics and presenteeism. Our findings reveal context‐specific mechanisms that extend the JD‐R theory and suggest potential intervention points for high‐reliability healthcare settings.

### 6.1 Strength and Limitations of the Work

The health impairment pathway was supported, showing that job stress was positively associated with burnout (*β* = 0.880, *p* < 0.001), which in turn accounted for 93% of its association with presenteeism. The motivational pathway revealed a clear hierarchy in resource efficacy: support utilization showed one of the largest protective indirect effects (*β*_indirect = −0.181, 80.1% mediated), substantially exceeding the effects of mere support availability (formal statistical comparisons were not conducted). In practical terms, a one‐standard‐deviation increase in support utilization (approximately 2 points) was associated with a 0.81‐point lower raw presenteeism score, which is a modest but statistically robust association.

These findings provide empirical grounding for the proposed ‘resource conversion bottleneck’, a theorized barrier that may prevent available support from being translated into utilized aid. Our results elaborate on its constituent mechanisms in three ways. First, the cognitive pathway is evidenced by the fact that support utilization (which requires active cognitive engagement) outperformed passively available support, suggesting that a high cognitive load in dialysis care may deplete the attentional resources needed for help‐seeking. Second, the cultural pathway is supported by the differential effects of support utilization versus objective support, suggesting that professional norms around autonomous expertise may create motivational barriers to seeking help, even when support structures exist. Third, the organizational pathway is reflected in the synergistic effects of structural factors (e.g., overtime and income), suggesting that fragmented workflows and time pressure may eliminate the ‘cognitive slack’ required to mobilize resources.

This construct is proposed as an explanatory lens for interpreting the observed hierarchy of resource effects, rather than as a directly measured mediator or moderator. Future empirical studies should directly measure this bottleneck construct to test its proposed mechanisms.

This bottleneck perspective extends the JD‐R and COR theories by specifying a conditional mechanism that has not been previously articulated: resource value depends not only on availability, but also on the individual's capacity and the work system's permission to overcome cognitive, cultural, and organizational barriers to access and deployment (Bakker and Demerouti 2018; Parker and Grote 2022). Thus, it offers a potential explanation for why intense demands in high‐reliability contexts may decouple resource availability from genuine utilization—a pattern observed here in the superior protective role of support utilization over mere availability.

The critical role of nurse staffing and the work environment in shaping both patient outcomes and nurse well‐being has been extensively documented and synthesized in recent evidence reviews (Griffiths et al. 2016). Our findings extend this knowledge by suggesting that the active utilization of support resources may be particularly critical within high‐reliability haemodialysis settings.

Our findings on the protective role of support utilization align with previous research suggesting positive associations between social support and nurses' work outcomes (Dong et al. 2023). The superior efficacy of support utilization over mere availability in our study extends this knowledge by specifying the conditions under which social support may be most beneficial.

Compared with nurses in other high‐risk healthcare settings, haemodialysis nurses face unique cognitive demands related to zero‐error tolerance and chronic exposure to critically ill patients. Nevertheless, the common challenges of burnout and presenteeism are shared across various healthcare populations. For instance, studies have documented a presenteeism prevalence of 43.8% among Spanish emergency and critical care nurses (Mu et al. 2026) and 58.4% among emergency nurses (Hou et al. 2025). Research has also shown that general practitioners are more likely to exhibit sickness presenteeism than other health professional groups (Pit et al. 2016), and correctional nurses experience elevated levels of burnout associated with their unique occupational environment, including high workloads and a lack of organizational support (Simpson et al. 2025). These findings suggest that while the specific work environment shapes the manifestation of occupational health challenges, the need for active resource mobilization—particularly support utilization—may be a universal protective mechanism across diverse health care contexts. Our findings on the 47.2% presenteeism prevalence among haemodialysis nurses are comparable to the global pooled estimate of 49.2% (95% CI: 41.1%–57.4%) reported in a meta‐analysis of 28 studies from 14 countries (Min et al. 2022). Similarly, a multi‐country integrative review noted that presenteeism rates in nursing vary widely (15.7%–86.9%) across different studies (Freeling et al. 2020). This international consistency suggests that presenteeism is not a regionally isolated phenomenon but a systemic issue facing the nursing workforce globally. However, cultural and organizational factors may influence the extent to which nurses translate available support into utilized aid, highlighting the need for cross‐cultural research on support‐utilization mechanisms.

Despite these contributions, this study has some limitations that should be considered. Its cross‐sectional design precludes definitive causal inferences about the JD‐R pathways; all reported associations should be interpreted as statistical relationships rather than causal effects. Reverse causality or unmeasured confounding variables could have influenced the observed associations. For example, nurses with higher presenteeism might perceive lower support utilization, or unmeasured personality factors could affect both resource perception and burnout levels. Self‐reported presenteeism is subject to social desirability and recall biases. Although the study was conducted in multiple provinces, the sample had a regional concentration, potentially limiting its generalizability. Furthermore, it should be noted that we did not conduct formal statistical comparisons (e.g., contrast analyses) to test the equivalence of the indirect effects across different resource dimensions. Therefore, any interpretation regarding the relative strength of specific resource dimensions should be considered exploratory. Future studies with larger samples are warranted to confirm this hierarchy of effects through formal statistical testing.

### 6.2 Recommendations for Further Research

Future research should:

Employ longitudinal or experience‐sampling methodologies to establish temporal precedence and explore the dynamics of resource conversion bottlenecks over time.

Conduct randomized controlled trials (RCTs) to test the efficacy of the proposed tiered intervention framework, particularly the combined effect of Resource Navigators and HRT simulations.

Incorporate objective or biometric measures (e.g., medication error rates, cortisol levels) to triangulate self‐reported presenteeism and burnout data.

Investigate these mechanisms in diverse cultural and healthcare systems to evaluate the generalizability of the resource conversion bottleneck concept.

### 6.3 Practical Implications: A Tiered Intervention Framework to Target Conversion Bottlenecks and Systemic Inequities

While systematic reviews have mapped the broad determinants of nurse presenteeism (Pereira et al. 2023), our study drills down to test the relative potency of specific resource dimensions, particularly the crucial step of utilization, within the defined high‐reliability context of haemodialysis. Our findings suggest a structured, four‐tier intervention framework for nursing management (see Appendix S4 in Supporting Information for detailed implementation protocols), moving from unit‐level process redesign to organizational policy reform. Each tier is designed to address specific barriers identified in our model.

#### 6.3.1 Tier 1: Enhance Resource Conversion Efficiency—Activating Support

##### 6.3.1.1 Core Action

Implement proactive support navigation systems where senior nurses act as resource navigators, using real‐time workload alerts to facilitate peer outreach (Sacco et al. 2020).

##### 6.3.1.2 Rationale

Shifts the initiation burden from cognitively depleted nurses to the support system, potentially accelerating support utilization and reducing emotional exhaustion.

#### 6.3.2 Tier 2: Engineer Safety Climate Synergies—Building Trust and Competence

##### 6.3.2.1 Core Action

Conduct mandatory, simulation‐based high‐reliability team training for dialysis‐specific emergencies, using structured communication protocols and debriefs (Barnes‐Daly et al. 2021).

##### 6.3.2.2 Rationale

Builds shared mental models, may reduce burnout‐inducing friction during crises, and leverages the unique direct protective association of trustworthy leadership identified in this study.

#### 6.3.3 Tier 3: Mitigate Core Job Stressors—Reducing Demand and Enabling Recovery

##### 6.3.3.1 Core Action

Deploy AI‐driven cognitive unloading tools (e.g., voice‐to‐text documentation, clinical decision support) to reduce extraneous cognitive load (Lee et al. 2023).

##### 6.3.3.2 Rationale

Directly addresses the cognitive depletion that may inhibit resource mobilization, potentially freeing attentional capacity for help‐seeking and patient care.

#### 6.3.4 Tier 4: Address Structural Factors—Considering Systemic Imbalances

##### 6.3.4.1 Core Action

Implement effort‐reward rebalancing policies, including specialty compensation premiums and evidence‐based overtime caps integrated with workload alerts (Siegrist 1996; Liu et al. 2023).

##### 6.3.4.2 Rationale

Directly targets the synergistic effects of low income and excessive overtime identified in our study, addressing foundational inequities that psychological interventions alone cannot remedy.

This tiered framework, from activating support to correcting structural inequities, offers a systematic approach to breaking the burnout–presenteeism cycle in high‐reliability nursing environments.

#### 6.3.5 Nursing Implications

The findings of this study offer several actionable implications for nursing managers in haemodialysis settings. First, building a help‐seeking culture is critical. Managers should shift the initiation burden from cognitively depleted nurses to the support system by designating senior nurses as resource navigators and using workload alerts to facilitate proactive peer outreach (Sacco et al. 2020). Second, fostering trustworthy leadership through visible management support and regular safety walk‐rounds can directly safeguard organizational performance and reduce presenteeism (Singer et al. 2023). Third, addressing structural inequities—such as implementing evidence‐based overtime caps and offering specialty compensation premiums—can mitigate the synergistic effects of low income and excessive overtime on burnout. These system‐level interventions are more sustainable than individual‐level coping strategies alone and should be integrated into routine nursing management practices to break the burnout–presenteeism cycle and enhance both nurse well‐being and patient safety (Liu et al. 2023).

## 7 Conclusion

In conclusion, this study supports an extended JD‐R model in haemodialysis nursing, identifying the proactive Utilization of Support as the resource most strongly associated with reduced burnout and presenteeism, and introducing the resource conversion bottleneck as a theoretical lens for understanding these patterns. However, due to the cross‐sectional design, these findings represent associations rather than causal relationships, and causal inferences cannot be drawn. Building on these findings, we suggest a tiered intervention framework targeting resource activation, safety culture engineering, cognitive demand reduction, and structural equity. These actions represent possible directions for supporting nurse well‐being and patient safety in high‐reliability care environments, though their effectiveness requires testing in future longitudinal and experimental studies.

## Author Contributions

**Xuejiao Li:** conceptualization, methodology, investigation, validation, formal analysis, software, writing – original draft, writing – review and editing, data curation, visualization. **Yan Zhang:** project administration, visualization. **Xia Liu:** investigation. **Wenxu Wang:** investigation. **Huili Jiang:** conceptualization, supervision, resources, funding acquisition.

## Funding

The authors have nothing to report.

## Disclosure

Xuejiao Li, the first author, completed all statistical analyses. She holds a Master of Nursing degree with advanced training in biostatistics and research methodology, and has extensive experience in structural equation modelling, mediation analysis (PROCESS macro), and multivariate statistics. The statistical methods employed—including bootstrapping (5000 resamples), false discovery rate correction, and hierarchical regression—were appropriately selected and correctly implemented. The authors affirm that the statistical findings are accurately reported and interpreted.

## Ethics Statement

This study received ethical approval from the Scientific Research Ethics Committee of Central Hospital Affiliated to Shandong First Medical University (Approval No.: 20241120008). All procedures performed were in accordance with the 1964 Helsinki declaration and its later amendments. Electronic informed consent was obtained from all individual participants prior to the study.

## Conflicts of Interest

The authors declare no conflicts of interest.

## Supporting information


**Data S1:** STROBE checklist.

**Appendix S1:** Pearson Correlation Matrix of all subscales (*N* = 583).

**Appendix S2:** Sensitivity analysis: FDR‐corrected significance of indirect effects.

**Appendix S3:** Detailed output of regression models testing interaction effects.

**Appendix S4:** Detailed implementation protocols for the tiered intervention framework.

## Data Availability

The data that support the findings of this study are available from the corresponding author upon reasonable request. The data are not publicly available due to privacy and ethical restrictions.
