# Supplementary material for: Support Utilization, Not Availability, Is Associated With Lower Burnout and Presenteeism Among Haemodialysis Nurses: A JD‐R Model Study
Source: Nurs Open. 2026 Oct 26;13(10):e70960. doi: 10.1002/nop2.70960 (PMC13710793; doi:10.1002/nop2.70960)
Supplement: Supplementary file 1 [file NOP2-13-e70960-s001.docx]

# **STROBE Statement—Checklist of items that should be included in reports of cross-sectional studies**

**Supplementary Material: STROBE Checklist**

| Item No | Recommendation | Page No |
| --- | --- | --- |
| ****Title and abstract**** |  |  |
| 1 | (a) Indicate the study’s design with a commonly used term in the title or the abstract | ****1**** |
|  | (b) Provide in the abstract an informative and balanced summary of what was done and what was found | ****1**** |
| ****Introduction**** |  |  |
| 2 | Background/rationale: Explain the scientific background and rationale for the investigation being reported | ****2-4**** |
| 3 | Objectives: State specific objectives, including any prespecified hypotheses | ****4-5**** |
| ****Methods**** |  |  |
| 4 | Study design: Present key elements of study design early in the paper | ****5**** |
| 5 | Setting: Describe the setting, locations, and relevant dates, including periods of recruitment, exposure, follow-up, and data collection | ****5-6**** |
| 6 | Participants: (a) Give the eligibility criteria, and the sources and methods of selection of participants | ****5-6**** |
| ****Variables**** |  |  |
| 7 | Clearly define all outcomes, exposures, predictors, potential confounders, and effect modifiers. Give diagnostic criteria, if applicable | ****6-7**** |
| ****Data sources/measurement**** |  |  |
| 8 | For each variable of interest, give sources of data and details of methods of assessment (measurement). Describe comparability of assessment methods if there is more than one group | ****6-7**** |
| ****Bias**** |  |  |
| 9 | Describe any efforts to address potential sources of bias | ****6-7**** |
| ****Study size**** |  |  |
| 10 | Explain how the study size was arrived at | ****5-6**** |
| ****Quantitative variables**** |  |  |
| 11 | Explain how quantitative variables were handled in the analyses. If applicable, describe which groupings were chosen and why | ****6-7**** |
| ****Statistical methods**** |  |  |
| 12 | (a) Describe all statistical methods, including those used to control for confounding | ****6**** |
|  | (b) Describe any methods used to examine subgroups and interactions | ****6**** |
|  | (c) Explain how missing data were addressed | ****6**** |
|  | (d) If applicable, describe analytical methods taking account of sampling strategy | ****N/A (Convenience sampling used)**** |
|  | (e) Describe any sensitivity analyses | ****14**** |
| ****Results**** |  |  |
| Participants |  |  |
| 13 | (a) Report numbers of individuals at each stage of study—eg numbers potentially eligible, examined for eligibility, confirmed eligible, included in the study, completing follow-up, and analysed | ****5-6**** |
|  | (b) Give reasons for non-participation at each stage | ****N/A (Anonymous survey; reasons not recorded)**** |
|  | (c) Consider use of a flow diagram | ****N/A (Details provided in text)**** |
| Descriptive data |  |  |
| 14 | (a) Give characteristics of study participants (eg demographic, clinical, social) and information on exposures and potential confounders | ****5–7, 9, 14–15**** |
|  | (b) Indicate number of participants with missing data for each variable of interest | ****6**** |
| Outcome data |  |  |
| 15 | Report numbers of outcome events or summary measures | ****7-14**** |
| Main results |  |  |
| 16 | (a) Give unadjusted estimates and, if applicable, confounder-adjusted estimates and their precision (eg, 95% confidence interval). Make clear which confounders were adjusted for and why they were included | ****11-17,**** |
|  | (b) Report category boundaries when continuous variables were categorized | ****6–7, 9**** |
|  | (c) If relevant, consider translating estimates of relative risk into absolute risk for a meaningful time period | N/A |
| Other analyses |  |  |
| 17 | Report other analyses done—eg analyses of subgroups and interactions, and sensitivity analyses | ****12-14**** |
| ****Discussion**** |  |  |
| Key results |  |  |
| 18 | Summarise key results with reference to study objectives | ****19–20**** |
| Limitations |  |  |
| 19 | Discuss limitations of the study, taking into account sources of potential bias or imprecision. Discuss both direction and magnitude of any potential bias | ****19–21**** |
| Interpretation |  |  |
| 20 | Give a cautious overall interpretation of results considering objectives, limitations, multiplicity of analyses, results from similar studies, and other relevant evidence | ****19–23**** |
| Generalisability |  |  |
| 21 | Discuss the generalisability (external validity) of the study results | ****19–21**** |
| ****Other information**** |  |  |
| Funding |  |  |
| 22 | Give the source of funding and the role of the funders for the present study and, if applicable, for the original study on which the present article is based | ****Title Page**** |
