# Supplementary material for: Support Utilization, Not Availability, Is Associated With Lower Burnout and Presenteeism Among Haemodialysis Nurses: A JD‐R Model Study
Source: Nurs Open. 2026 Oct 26;13(10):e70960. doi: 10.1002/nop2.70960 (PMC13710793; doi:10.1002/nop2.70960)
Supplement: Supplementary file 2 [file NOP2-13-e70960-s002.docx]

### ****Appendix C. Detailed Output of Regression Models Testing Interaction Effects****

****Table C1.**** Hierarchical Regression Analysis Testing the Interaction Effect of Monthly Income and Overtime Frequency on Burnout (MBI Score)

| **Predictor** | **Model 1: Main Effects Only** |  |  |  | **Model 2: Main Effects + Interaction** |  |  |  |
| --- | --- | --- | --- | --- | --- | --- | --- | --- |
|  | ***B*** | ***SE*** | ***t*** | ***p*** | ***B*** | ***SE*** | ***t*** | ***p*** |
| **(Constant)** | 44.85 | 7.11 | 6.31 | <0.001 | 43.12 | 7.02 | 6.14 | <0.001 |
| **Overtime (days/week)** | 4.00 | 0.87 | 4.60 | <0.001 | 3.85 | 0.85 | 4.53 | <0.001 |
| **Monthly Income (Ref: >10000 RMB)** |  |  |  |  |  |  |  |  |
| → ≤4000 RMB | 1.91 | 0.52 | 3.67 | <0.001 | 1.78 | 0.51 | 3.49 | 0.001 |
| → 4001–8000 RMB | 0.49 | 2.75 | 0.18 | 0.858 | 0.41 | 2.71 | 0.15 | 0.880 |
| → 8001–10000 RMB | -4.81 | 4.08 | -1.18 | 0.239 | -4.90 | 4.02 | -1.22 | 0.224 |
| **Interaction Term** |  |  |  |  |  |  |  |  |
| → **Overtime × Income (≤4000 RMB)** |  |  |  |  | **6.92** | **1.24** | **5.58** | **<0.001** |
| **Model Fit** |  |  |  |  |  |  |  |  |
| R² | 0.38 |  |  |  | 0.45 |  |  |  |
| ΔR² | - |  |  |  | 0.07 |  |  |  |
| F for ΔR² (df1, df2) | - |  |  |  | 31.15** (1, 562) |  |  |  |

Note: *B* = unstandardized coefficient; SE = standard error. The interaction term is between the dummy-coded variable for Income ≤4000 RMB and the continuous Overtime variable. The significant positive *B* coefficient for the interaction indicates that the combination of low income and high overtime frequency predicts significantly higher levels of burnout than would be expected by adding their individual effects.

****Table C2.**** Hierarchical Regression Analysis Testing the Interaction Effect of Self-Rated Health and Organizational Support on Presenteeism (SPS-6 Score)

| **Predictor** | **Model 1: Main Effects Only** |  |  |  | **Model 2: Main Effects + Interaction** |  |  |  |
| --- | --- | --- | --- | --- | --- | --- | --- | --- |
|  | ***B*** | ***SE*** | ***t*** | ***p*** | ***B*** | ***SE*** | ***t*** | ***p*** |
| **(Constant)** | 11.65 | 1.40 | 8.33 | <0.001 | 11.70 | 1.38 | 8.48 | <0.001 |
| **Self-Rated Health (Ref: Good)** |  |  |  |  |  |  |  |  |
| → Poor | 3.25 | 0.47 | 6.91 | <0.001 | 2.10 | 0.52 | 4.04 | <0.001 |
| → Fair | 0.84 | 0.34 | 2.50 | 0.013 | 0.79 | 0.33 | 2.39 | 0.017 |
| **Org. Support (Ref: Provided)** |  |  |  |  |  |  |  |  |
| → Not Provided | 1.27 | 0.44 | 2.89 | 0.004 | 1.05 | 0.43 | 2.44 | 0.015 |
| **Interaction Term** |  |  |  |  |  |  |  |  |
| → **Health (Poor) × Support (Not Provided)** |  |  |  |  | **4.31** | **0.59** | **7.32** | **<0.001** |
| **Model Fit** |  |  |  |  |  |  |  |  |
| R² | 0.43 |  |  |  | 0.48 |  |  |  |
| ΔR² | - |  |  |  | 0.05 |  |  |  |
| F for ΔR² (df1, df2) | - |  |  |  | 53.59** (1, 561) |  |  |  |

Note: The significant positive B coefficient for the interaction indicates that nurses who reported both poor health and a lack of organizational support had significantly higher presenteeism scores than the sum of the individual effects of these two risk factors.

****Table C3.**** Hierarchical Regression Analysis Testing the Moderating Effect of Family Attitude on the Relationship Between Burnout and Presenteeism

| **Predictor** | **Model 1: Main Effects Only** |  |  |  | **Model 2: Main Effects + Interaction** |  |  |  |
| --- | --- | --- | --- | --- | --- | --- | --- | --- |
|  | ***B*** | ***SE*** | ***t*** | ***p*** | ***B*** | ***SE*** | ***t*** | ***p*** |
| **(Constant)** | 11.65 | 1.40 | 8.33 | <0.001 | 11.60 | 1.39 | 8.35 | <0.001 |
| **Burnout (MBI Score)** | 0.105 | 0.010 | 10.50 | <0.001 | 0.098 | 0.010 | 9.80 | <0.001 |
| **Family Attitude (Ref: Supportive)** |  |  |  |  |  |  |  |  |
| → Unsupportive | 2.05 | 0.51 | 4.02 | <0.001 | 1.80 | 0.51 | 3.53 | 0.001 |
| → Neutral | 1.69 | 0.87 | 1.94 | 0.053 | 1.55 | 0.86 | 1.80 | 0.072 |
| **Interaction Term** |  |  |  |  |  |  |  |  |
| → **Burnout × Attitude (Unsupportive)** |  |  |  |  | **0.068** | **0.025** | **2.72** | **0.008** |
| **Model Fit** |  |  |  |  |  |  |  |  |
| R² | 0.43 |  |  |  | 0.44 |  |  |  |
| ΔR² | - |  |  |  | 0.01 |  |  |  |
| F for ΔR² (df1, df2) | - |  |  |  | 7.38** (1, 561) |  |  |  |

Note: The significant positive *B* coefficient for the interaction indicates that the relationship between burnout and presenteeism is stronger for nurses who report unsupportive family attitudes compared to those with supportive families. Burnout has a more detrimental effect on presenteeism when family support is lacking.
