# Supplementary material for: Support Utilization, Not Availability, Is Associated With Lower Burnout and Presenteeism Among Haemodialysis Nurses: A JD‐R Model Study
Source: Nurs Open. 2026 Oct 26;13(10):e70960. doi: 10.1002/nop2.70960 (PMC13710793; doi:10.1002/nop2.70960)
Supplement: Supplementary file 3 [file NOP2-13-e70960-s003.docx]

#### **Appendix D. Detailed Implementation Protocols for the Tiered Intervention Framework**

This appendix provides the detailed operational protocols for the four-tier intervention framework outlined in the main text (Section 6.3). These protocols are designed for nursing managers and healthcare administrators seeking to implement evidence-based strategies to reduce burnout and presenteeism among hemodialysis nurses.

### **Tier 1: Enhance Resource Conversion Efficiency – Activating Support**

**Core Action:** Implement a **Proactive Support Navigation System**

****1.1. Resource Navigator Role****

**Position:** Designate one senior nurse per shift (minimum 5 years hemodialysis experience) as the "Resource Navigator."

**Time Allocation:** Allocate 4–6 hours per week of protected, non-clinical time for navigator duties.

**Training:** Provide 8-hour initial training covering:

Recognition of early burnout signs in colleagues

Peer support communication techniques (active listening, non-judgmental approach)

Confidentiality protocols and escalation pathways

****1.2. Workload Alert System****

**Data Integration:** Connect the alert system to the Electronic Health Record (EHR) to capture real-time indicators:

Patient acuity scores (e.g., Acuity Level 3+ patients per nurse > 3)

Overtime accumulation (>2 hours of overtime in a single shift)

Missed break alerts (no 30-minute break recorded after 6 hours)

**Alert Thresholds:** System generates automated alerts to the Resource Navigator when:

Any nurse exceeds 2 of the above thresholds in a single shift

A specific unit area shows >50% of nurses exceeding thresholds

****1.3. Proactive Outreach Protocol****

**Trigger:** Upon receiving an alert, the Resource Navigator conducts a brief (5–10 minute) check-in with the identified nurse(s).

**Check-in Content:**

"How are you managing the workload today?"

"Is there one task I can help with right now to ease the pressure?"

"Have you taken your break? Can I cover for 15 minutes while you step away?"

**Escalation:** If the nurse reports feeling overwhelmed, the Navigator can:

Reallocate one non-urgent task to themselves or another available staff member

Authorize an additional 15-minute break

Refer to Employee Assistance Program (EAP) if emotional distress is evident

****1.4. Documentation and Feedback****

Log all outreach encounters in a secure, de-identified database (tracking only: date, shift, reason for alert, action taken).

Monthly review of logs by nurse manager to identify patterns and adjust staffing as needed.

### **Tier 2: Engineer Safety Climate Synergies – Building Trust and Competence**

**Core Action:** Conduct **High-Reliability Team (HRT) Simulation Training**

****2.1. Simulation Schedule****

**Frequency:** Quarterly (4 sessions per year)

**Duration:** 3 hours per session (including setup, simulation, debrief)

**Participants:** Interprofessional triads (1 nurse, 1 physician, 1 dialysis technician) – rotate participants to ensure all staff attend at least 2 sessions per year.

****2.2. Simulation Scenarios****
Develop and rotate between three dialysis-specific emergency scenarios:

| **Scenario** | **Description** | **Key Skills Tested** |
| --- | --- | --- |
| Scenario A: Cardiac Arrest on Dialysis | Patient develops ventricular fibrillation during treatment | Early recognition, CPR coordination, defibrillator use, role delegation |
| Scenario B: Severe Air Embolism | Air detected in venous line, patient in distress | Immediate line clamping, trendelenburg positioning, oxygen administration, team communication |
| Scenario C: Anaphylactic Reaction | Patient develops severe reaction to dialyzer or medication | Rapid assessment, medication administration, airway management, family communication |

****2.3. Structured Communication Protocol (SBAR)****
All simulations require use of SBAR format for all handoffs and escalation:

| Component | Description | Example |
| --- | --- | --- |
| **S**ituation | What is happening now? | "Patient is in cardiac arrest, CPR in progress." |
| **B**ackground | Relevant clinical context | "Patient is 65-year-old on dialysis for 3 years, no prior cardiac history." |
| **A**ssessment | What you think the problem is | "Possible hyperkalemia-induced arrest." |
| **R**ecommendation | What you need | "Need crash cart and code team immediately." |

****2.4. Video-Assisted Debriefing****

**Recording:** Record all simulations (with participant consent, footage used only for internal education, deleted after debrief).

**Debrief Structure (20 minutes):**

**Reaction Phase (5 min):** "How did that feel? What went well?"

**Analysis Phase (10 min):** Play key video segments – focus on communication breakdowns or successes.

**Summary Phase (5 min):** "What will we do differently next time?"

**Facilitator:** Trained simulation facilitator (can be senior nurse or educator) guides debrief using advocacy-inquiry approach.

****2.5. Competency Tracking****

Maintain a log of staff participation.

Annual competency sign-off for emergency response based on simulation performance.

### **Tier 3: Mitigate Core Job Stressors – Reducing Demand and Enabling Recovery**

**Core Action:** Deploy **AI-Driven Cognitive Unloading Tools**

****3.1. Voice-to-Text Documentation System****

**Technology:** Implement a Natural Language Processing (NLP) system integrated with the EHR (e.g., a commercial voice-to-text system, or similar).

**Functionality:** Nurses can dictate:

Patient assessments

Shift notes

Incident reports

**Accuracy Protocol:** System provides real-time transcription with 95%+ accuracy; nurse reviews and edits before signing.

**Training:** 2-hour individual training session for all nurses; ongoing tech support available.

****3.2. Automated SOAP Note Structuring****

**Workflow Integration:** After voice dictation, the AI system automatically organizes content into SOAP format:

**S**ubjective: Patient-reported symptoms (e.g., "patient reports feeling dizzy")

**O**bjective: Vital signs, physical assessment findings

**A**ssessment: Nurse's clinical judgment (e.g., "likely intradialytic hypotension")

**P**lan: Next steps (e.g., "hold antihypertensive, monitor BP q15min")

**Time Savings Target:** ≥25% reduction in documentation time (based on Zhou et al., 2023).

****3.3. Clinical Decision Support Alerts****

**Heparin Verification:** AI cross-checks heparin dose against patient weight, lab results (aPTT), and recent doses; flags discrepancies before administration.

**Fluid Removal Calculation:** AI calculates recommended ultrafiltration rate based on patient's estimated dry weight and current fluid status; alerts if prescribed rate exceeds safe threshold.

**Medication Interaction Check:** Real-time alert if newly ordered medication interacts with current dialysis prescription.

****3.4. Implementation Timeline****

| Phase | Activities | Duration |
| --- | --- | --- |
| Phase 1: Vendor Selection | Evaluate 3-4 AI documentation systems; select based on EHR compatibility and nursing input | 2 months |
| Phase 2: Technical Integration | IT team integrates selected system with existing EHR; conduct security and privacy review | 2 months |
| Phase 3: Pilot Testing | Test with 10 volunteer nurses for 4 weeks; collect feedback and adjust | 1 month |
| Phase 4: Full Rollout | Train all nurses; go-live with IT support available 24/7 for first 2 weeks | 1 month |
| Phase 5: Evaluation | Measure documentation time, satisfaction, and error rates at 3 and 6 months post-implementation | Ongoing |

### **Tier 4: Address Structural Factors – Considering Systemic Imbalances**

**Core Action:** Implement **Effort-Reward Rebalancing Policies**

****4.1. Hemodialysis Specialty Compensation Premium****

**Policy:** Establish a tiered compensation structure recognizing specialty skills and high-acuity work.

**Premium Rates:**

Base: 100% of general ward nurse salary

Hemodialysis-certified nurses: **115%** of base

Hemodialysis-certified with ≥5 years experience: **120%** of base

Charge nurses/shift coordinators: Additional 5% differential

**Implementation:** Phased approach over 2 years to allow budget adjustment.

****4.2. Evidence-Based Overtime Cap****

**Cap:** Maximum **8 hours of overtime per month** per nurse (excluding emergencies declared by hospital administration).

**Workload Alert Integration:** When a nurse approaches 6 hours of overtime in a month, their manager receives an alert to:

Review upcoming schedule

Offer a compensatory day off within the next 2 weeks

Adjust future shift assignments to prevent exceeding the cap

**Emergency Exemption:** In declared emergencies (e.g., pandemic surge, natural disaster), the cap may be temporarily suspended with:

Written justification from nursing director

Mandatory 48-hour rest period after any 12+ hour shift

Double pay for all hours exceeding the cap

****4.3. Staffing Ratio Standards****

**Target Nurse-to-Patient Ratio:** 1:3 for acute hemodialysis; 1:4 for chronic outpatient hemodialysis (based on acuity).

**Acuity-Based Adjustments:**

Patients requiring continuous monitoring (e.g., unstable vitals, new dialysis starts): 1:2

Patients with complex needs (e.g., multiple comorbidities, language barriers): 1:3

Stable, long-term patients: 1:4

**Compliance Monitoring:** Monthly audit of staffing ratios; any deviation >10% requires written explanation from nursing management.

****4.4. Wellness and Recovery Support****

**On-Site Wellness Room:** Dedicated, quiet space (not a break room) with comfortable seating, dim lighting, and noise reduction – available 24/7.

**Peer Support Program:** Trained peer supporters available for confidential conversations; 1-hour initial training plus monthly supervision.

**EAP Enhancement:** Ensure EAP counselors have specific training in healthcare worker trauma and burnout; offer 8 free sessions per issue (increased from standard 4).

### **Implementation Timeline Summary**

| Timeline | Tier 1 | Tier 2 | Tier 3 | Tier 4 |
| --- | --- | --- | --- | --- |
| **Months 1-3** | Select Resource Navigators; develop alert system | Plan simulation scenarios; train facilitators | Vendor selection; IT integration planning | Budget planning for premium; draft policy |
| **Months 4-6** | Pilot alert system; train navigators | Pilot first simulation session | Pilot voice-to-text with volunteers | Present premium proposal to hospital leadership |
| **Months 7-9** | Full rollout of navigation system | Quarterly simulations begin | Full AI tool rollout | Implement premium for certified nurses |
| **Months 10-12** | Monthly log review and adjustment | Expand scenarios; track participation | Evaluate documentation time savings | Implement overtime cap; monitor compliance |
| **Year 2+** | Annual refresher training for navigators | Annual competency sign-off | Ongoing system updates | Review premium impact on retention |

**Note:** All protocols should be adapted to local hospital policies, resources, and regulatory requirements. Implementation should involve frontline nursing staff in planning and decision-making to ensure buy-in and contextual relevance. Outcome evaluation (burnout, presenteeism, retention) should be conducted at 12 and 24 months post-implementation.
