# Supplementary material for: Support Utilization, Not Availability, Is Associated With Lower Burnout and Presenteeism Among Haemodialysis Nurses: A JD‐R Model Study
Source: Nurs Open. 2026 Oct 26;13(10):e70960. doi: 10.1002/nop2.70960 (PMC13710793; doi:10.1002/nop2.70960)
Supplement: Supplementary file 4 [file NOP2-13-e70960-s004.docx]

#### ****Appendix B. Sensitivity Analysis: FDR-Corrected Significance of Indirect Effects****

| Indirect Path (X → M → Y) | Indirect Effect (*β*) | Boot SE | Original 95% CI | Original *p*-value | FDR-Adjusted *p*-value | Significant after FDR? (*α* = 0.05) |
| --- | --- | --- | --- | --- | --- | --- |
| Objective Support → Burnout → Presenteeism | -0.877*** | 0.191 | [-1.29, -0.54] | < 0.001 | 0.0012 | Yes |
| Subjective Support → Burnout → Presenteeism | -0.117*** | 0.018 | [-0.15, -0.08] | < 0.001 | 0.0012 | Yes |
| **Support Utilization → Burnout → Presenteeism** | **-0.181*** | **0.020** | **[-0.22, -0.14]** | **< 0.001** | **0.0012** | **Yes** |
| Teamwork Climate → Burnout → Presenteeism | -0.204*** | 0.023 | [-0.25, -0.16] | < 0.001 | 0.0012 | Yes |
| Management Perceptions → Burnout → Presenteeism | -0.201*** | 0.020 | [-0.24, -0.16] | < 0.001 | 0.0012 | Yes |
| Job Stress → Burnout → Presenteeism | 0.093* | 0.013 | [0.07, 0.12] | 0.018 | 0.0216 | Yes |

**Note:** FDR correction was applied using the Benjamini-Hochberg procedure across all 6 indirect paths. All indirect effects remained significant after FDR adjustment, confirming the robustness of the mediation findings.
