# Supplementary material for: Support Utilization, Not Availability, Is Associated With Lower Burnout and Presenteeism Among Haemodialysis Nurses: A JD‐R Model Study
Source: Nurs Open. 2026 Oct 26;13(10):e70960. doi: 10.1002/nop2.70960 (PMC13710793; doi:10.1002/nop2.70960)
Supplement: Supplementary file 5 [file NOP2-13-e70960-s005.docx]

### ****Appendix A. Pearson Correlation Matrix of All Subscales (*N* = 583)****

| Variable | 1 | 2 | 3 | 4 | 5 | 6 | 7 | 8 | 9 | 10 | 11 | 12 | 13 | 14 | 15 | 16 | 17 | 18 |
| --- | --- | --- | --- | --- | --- | --- | --- | --- | --- | --- | --- | --- | --- | --- | --- | --- | --- | --- |
| ****1. Presenteeism (Total)**** | 1 |  | . |  |  |  |  |  |  |  |  |  |  |  | . | . | . |  |
| ****2. Presenteeism (Work Impairment)**** | .849** | 1 |  |  |  |  |  |  |  |  |  |  |  |  |  |  |  |  |
| ****3. Presenteeism (Work Energy)**** | .337** | –.212** | 1 |  |  |  |  |  |  |  |  |  |  |  |  |  |  |  |
| ****4. Social Support (Total)**** | –.327** | –.301** | –.069 | 1 |  |  |  |  |  |  |  |  |  |  |  |  |  |  |
| ****5. Social Support (Objective)**** | –.239** | –.211** | –.067 | .804** | 1 |  |  |  |  | . |  |  |  |  |  |  |  |  |
| ****6. Social Support (Subjective)**** | –.318** | –.284** | –.081 | .897** | .529** | 1 |  |  |  |  |  |  |  |  |  |  |  |  |
| ****7. Social Support (Utilization)**** | –.171** | –.195** | .031 | .586** | .345** | .359** | 1 |  |  |  |  |  |  |  |  |  |  |  |
| ****8. Safety Attitudes (Total)**** | –.432** | –.384** | –.116** | .335** | .242** | .308** | .224** | 1 |  |  |  |  |  |  |  |  |  |  |
| ****9. SAQ Teamwork Climate**** | –.380** | –.334** | –.107** | .300** | .201** | .280** | .218** | .865** | 1 |  |  |  |  |  |  |  |  |  |
| ****10. SAQ Safety Climate**** | –.363** | –.313** | –.113** | .286** | .205** | .255** | .216** | .894** | .804** | 1 |  | . |  |  |  |  |  |  |
| ****11. SAQ Management Perceptions**** | –.344** | –.286** | –.127** | .229** | .176** | .208** | .142** | .885** | .739** | .807** | 1 |  |  |  |  |  |  |  |
| ****12. SAQ Job Satisfaction**** | –.372** | –.332** | –.095* | .300** | .218** | .289** | .167** | .883** | .683** | .738** | .821** | 1 |  |  |  |  |  |  |
| ****13. SAQ Working Conditions**** | –.336** | –.293** | –.098* | .246** | .182** | .217** | .181** | .870** | .697** | .757** | .798** | .840** | 1 |  |  |  |  |  |
| ****14. SAQ Stress Recognition**** | –.141** | –.156** | .017 | .127** | .098* | .119** | .066 | .087* | –.053 | –.126** | –.149** | –.109** | –.182** | 1 |  |  |  |  |
| ****15. Burnout (Total)**** | .595** | .592** | .045 | –.401** | –.317** | –.357** | –.254** | –.578** | –.514** | –.483** | –.404** | –.506** | –.457** | –.210** | 1 |  |  |  |
| ****16. Burnout (Emotional Exhaustion)**** | .493** | .591** | –.140** | –.296** | –.220** | –.272** | –.190** | –.380** | –.282** | –.262** | –.231** | –.372** | –.308** | –.239** | .803** | 1 |  |  |
| ****17. Burnout (Depersonalization)**** | .461** | .522** | –.077 | –.307** | –.235** | –.265** | –.229** | –.398** | –.376** | –.343** | –.268** | –.350** | –.314** | –.116** | .752** | .676** | 1 |  |
| ****18. Burnout (Reduced Accomplishment)**** | .304** | .154** | .288** | –.249** | –.217** | –.217** | –.134** | –.437** | –.439** | –.422** | –.350** | –.337** | –.336** | –.061 | .555** | .001 | .087* | 1 |

**Note:** *N* = 583 for all correlations.* *p* < .05, ** *p* < .01 (two-tailed). Only the lower triangle of the correlation matrix is shown for clarity. SPS-6 = Stanford Presenteeism Scale-6; MBI = Maslach Burnout Inventory; SSRS = Social Support Rating Scale; SAQ = Safety Attitudes Questionnaire.
